# Coordinating Calvin Cycle and Glycolysis in *Escherichia coli*

**DOI:** 10.1021/acssynbio.5c00854

**Published:** 2026-03-26

**Authors:** Yu-Jen Lin, Hsien-Tse Chen, Pei-Yi Lin, Yi-Jyun Lai, Si-Yu Li

**Affiliations:** Department of Chemical Engineering, 34916 National Chung Hsing University, Taichung 402, Taiwan

**Keywords:** Calvin cycle, glycolysis, Escherichia coli, central metabolism rewiring, mixotroph, mass-action rebalancing

## Abstract

Engineering *Escherichia coli* to coassimilate glucose and CO_2_ requires rewiring central metabolism so a Calvin-Benson-Bassham (CBB) module can compete with, rather than be overwhelmed by, glycolysis. We implemented a Rubisco-based engineered pathway, i.e., heterologous phosphoribulokinase (PrkA) and ribulose-1,5-bisphosphate carboxylase/oxygenase (Rubisco), and attenuated glycolysis by (i) CRISPRi repression of *gapA* (GAPDH) and (ii) deletion of major fermentative redox sinks (Δ*ldhA* Δ*frd*). Activation of the Rubisco-based engineered pathway not only enabled CO_2_ fixation but also unexpectedly revived glycolytic throughput, yielding a coordinated “harmony” between the two pathways. The engineered strain (*E. coli* MZLF/pSLiP, pCCS01) sustained cometabolism, consuming glucose at 170 ± 1 mg L^–1^ h^–1^ by 84 h. Flux analysis indicated that 17.0 ± 0.1 mg L^–1^ h^–1^, 10% of total glucose uptake, was routed through the Rubisco-based engineered pathway, corresponding to a CO_2_-fixation rate of 5.0 ± 0.1 mg L^–1^ h^–1^. Mechanistically, in the parental *E. coli* background (*ldhA* ^+^, *frd* ^+^) ATP is primarily supplied by glycolysis with redox balance via lactate/succinate formation. With the CBB module active in the engineered context (*gapA* repressed, Δ*ldhA* Δ*frd*, *prkA* ^+^, *rbcLS* ^+^), pyruvate allocation, ATP from acetate, and NADH reoxidation from ethanol production jointly determine flux partitioning, yielding a distinct fermentation profile. These findings show that successful central-metabolism rewiring must target not only core nodes (e.g., *gapA*) but also auxiliary redox circuits (*ldhA*, *frd*). Equally important is maintaining a moderate activity of the Rubisco-based engineered pathway, which allows restoration of the near-equilibrium state at the GAPDH-repressed G3*P*/1,3-BPG/3PG node.

## Graphical abstract

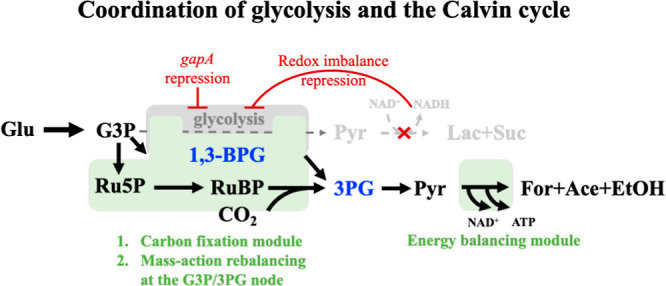

## 1 Introduction

In nature, metabolism can be either autotrophic or heterotrophic. Both strategies coexist in mutualistic relationships and confer evolutionary advantages. Autotrophic metabolism is primarily accomplished through photosynthesis in plants, as well as by photoautotrophic and chemoautotrophic microorganisms.^1^ The Calvin-Benson-Bassham (CBB) cycle is the dominant carbon assimilation route on Earth, responsible for over 99.5% of carbon fixation reactions.^2^ This cycle involves 11 catalytic enzymes, most of which also participate in central metabolism of glycolysis and the pentose phosphate pathway. Two signature enzymes clearly define the pathway: phosphoribulokinase (PrkA) and ribulose-1,5-bisphosphate carboxylase/oxygenase (Rubisco).^3^ When engineered as a Rubisco-based pathway in *Escherichia coli*, the carboxylation reaction enables the fixation of CO_2_ into the cell, forming the essential core of biological carbon assimilation.^4^

Heterotrophic organisms rely on organic compounds as both carbon and energy sources. Glucose is one of the most common and chemically versatile molecules, serving as a central node in cellular metabolism. Its catabolism through glycolysis not only provides ATP but also generates reducing equivalents and metabolic intermediates essential for biosynthesis. Glycolysis (Embden-Meyerhof-Parnas pathway, EMP), together with auxiliary pathways such as the pentose phosphate pathway (PPP), and the Entner-Doudoroff (ED) pathway comprises the glycolytic routes. *E. coli* exemplifies the heterotrophic lifestyle and serves as a model organism for studying glucose metabolism. Its core metabolic network integrates glycolysis, the PPP, the ED pathway, the TCA cycle, and several fermentative routes. Due to the efficiency of glycolysis as evolutionary advantage, *E. coli* primarily depends on this pathway to meet its energy demand under glucose-fed conditions.

Rewiring central metabolism provides opportunities to overcome physiological bottlenecks and enable novel engineering applications. For example, dynamic regulation of glucose entry into glycolysis has been proposed to attenuate the Crabtree effect in *Saccharomyces cerevisiae*, thereby reducing ethanol overflow and redirecting carbon toward high-value biosynthesis.^5^ Our previous work in metabolic engineering have demonstrated the potential of incorporating Rubisco and PrkA into *E. coli* for in situ CO_2_ recycling and yield enhancement using glucose as feedstock. Heterologous expression of PrkA and Rubisco enables CO_2_ fixation during fermentation, reducing carbon loss and redirecting flux into central metabolism.^6^ To further channel carbon toward this pathway, *zwf* (encoding glucose-6-phosphate dehydrogenase) was deleted, thereby blocking the oxidative pentose phosphate pathway and forcing flux through the nonoxidative branch, which supplies ribulose-5-phosphate as the substrate for PrkA-Rubisco cycling. This modification, together with the use of the C2/C1 ratio as a practical metric, provided a robust framework to evaluate in situ CO_2_ recycling efficiency.^7^ Building on this foundation, mixotrophic designs combining Rubisco, *zwf* deletion, and a pyruvate-to-ethanol “carbon tap valve” exceeded conventional theoretical fermentation yields, achieving product yields above the canonical 2.0 mol per mol glucose.^8^ Our previous study also demonstrates that Rubisco activity is subject to RuBP inhibition, but can be relieved through coexpression of Rubisco activase, underscores the importance of enzyme regulation in sustaining high catalytic efficiency and maximizing CO_2_ utilization.^9^

Mixotrophic growth offers high growth rates while having substantial CO_2_ fixation capacity. Although pentoses can feed the nonoxidative PPP to generate Ru5P for PrkA and thereby support Rubisco-mediated fixation,^10–12^ their cost often limits practicality. Glucose is inexpensive and readily available, making it the most practical cofeedstock. However, because glycolysis is ubiquitous and evolutionary favored, competition glycolysis and CO_2_ fixation must be managed in glucose-driven CO_2_ fixation. Besides, a molecular understanding of how autotrophic pathways can be integrated and functionally coexist with heterotrophic metabolism remains largely unexplored.

To enable glucose-driven CO_2_ fixation in minimal medium, glycolysis was first attenuated by CRISPRi-mediated repression of *gapA* (encoding glyceraldehyde-3-phosphate dehydrogenase), and this attenuation was reinforced by double deletions of *ldhA* (encoding lactate dehydrogenase) and *frd* (encoding fumarate reductase). Under these conditions, restoration of balanced glucose metabolism and competitiveness with glycolysis by reconstituted Calvin cycle was evaluated, and glycolytic flux partition was quantified by LC-QTOF-based ^13^C tracing, thereby validating mixotrophic growth and product formation. The gene *gapA* was targeted to suppress glycolysis because its deletion also blocks flux through the Entner-Doudoroff pathway, which produces glyceraldehyde-3-phosphate, the substrate of GapA. Although ED activity is weak in *E. coli*,^13^ this strategy ensures comprehensive attenuation of glucose catabolism.

## 2 Materials and Methods

### 2.1 Bacterial Strain and Plasmid Construction

All bacterial strains and plasmids used in this study are listed in Table 1.

**Table 1:** Strains and Plasmids Used in This Study

strain/plasmid	descriptions	ref
strains		
E. coli DH5α	*FhuA2*Δ*(argF-lacZ)U169 phoA gln V44 Φ80*Δ*(lacZ)M15 gyr A96 recA1 relA1 endA1 thi-1 hsdR17*	lab stock
E. coli BL21(DE3)	F^–^ *OmpT* *gal dcm lon hsdSB*(*rB* ^–^ *mB* ^–^) λ(DE3)	lab stock
E.coli MZ	E.coli BL21(DE3)Δ*zwf*	lab stock
E.coli MZL	E.coli BL21(DE3)Δ*zwf*Δ*ldh*	lab stock
E.coli MZLF	E.coli BL21(DE3)Δ*zwf*Δ*ldh*Δ*frd*	lab stock
plasmids		
pBAD-his6-prkA-pACYC184	Low-copy plasmid carrying *prkA* with an N-terminal His6-tag under the arabinose-inducible P_BAD_; confers chloramphenicol resistance and enables regulated PrkA expression in E. coli	(3)
*rbcLS*-pET30a+(M259T)	carrying *rbcL* and *rbcS* under the T7 promoter; confers kanamycin resistance and enables high-level Rubisco expression in E. coli BL21(DE3)	(3)
pSLiP-G2-RacE	RK2 ori, Km^r^, containing J23119 promoter with PAM, l-rhamnose inducible dCas9, J23114-*racE* gene and sgRNA-G2	(Kim et al. 2022)
pCCS01	Plasmid assembly was performed by joining *Xba*I/*Hind*III-linearized pBAD-his6-prkA-pACYC184 (vector backbone) with a PCR-linearized *rbcLS* insert derived from *rbcLS*-pET30a+(M259T). In the resulting construct, *prkA* is driven by the P_BAD_ promoter, whereas *rbcLS* is under the T7 promoter	this study

Recombinant plasmids were constructed and maintained in *E. coli* DH5α. Transformation was performed using electroporation (Bio-Rad Gene Pulser, Bio-Rad, California, USA). The recombinant plasmid pCCS01 was obtained by construction involving two plasmids, pBAD-his6-prkA-pACYC184 and rbcLS-pET30a­(+). The former served as the vector and was linearized using the restriction enzymes *Hind*III and *Xba*I (New England BioLabs Inc., MA, USA), containing the *prkA* gene. From the latter, the *rbcLS* fragment was amplified using Q5 High Fidelity DNA Polymerase (New England BioLabs Inc., MA, USA) with primers HF-R-rbcLS-CCS01 and HF-L-rbcls-CCS01. The linearized vector and amplified insert were assembled by NEBuilder HiFi DNA Assembly (New England BioLabs Inc., MA, USA) to generate the recombinant plasmid pCCS01. Transformed colonies were selected on LB agar plates supplemented with chloramphenicol, and positive clones were verified by PCR using the primers prkA-lpp-r, CCS01-R-CK-1, and seq-F-rbcLS-02. The whole sequence of pCCS01 was confirmed by Sanger sequencing. Table 2 list primers used in this study.

**Table 2:** Primers Used in This Study

primer	sequence (5′ → 3′)
HF–F-rbcLS-CCS01	ttttcagcttgttgactctaGGTGCCTAATGAGTGAGC
HF-R-rbcLS-CCS01	ttattaatcagataaaatatttCAATCCGGATATAGTTCCTC
prkA-lpp-r	AGCATTCTGTAACAAAGCGG
CCS01-R-CK-1	CCTGCAAGGCGGTTTTTTCG
seq-F-rbcLS-02	TCACTGCCCGCTTTCCAGTC

### 2.2 Bacterial Cultivation

Precultures were grown in LB medium at 37 °C in a rotary shaker (200 rpm) for 15 h. For anaerobic batch cultivation, 250 mL serum bottles sealed with butyl rubber stoppers and aluminum crimps were sterilized by autoclaving at 121 °C and 1.2 kg/cm^2^ for 30 min. A 5× M9 salts solution (33.9 g/L NaHPO_4_, 15 g/L KH_2_PO_4_, 2.5 g/L NaCl, 5.0 g/L NH_4_Cl) and a 400 g/L glucose stock were sterilized by autoclaving, while a 100× trace element solution (200 mM MgSO_4_, 10 mM CaCl_2_) was sterilized by syringe filtration. These three solutions were subsequently added to the 250 mL serum bottles, together with the appropriate antibiotics, and diluted to a final working volume of 50 mL.

To establish anaerobic conditions, the bottle headspace was sparged with CO_2_ for 10 min using a needle inserted through the rubber stopper to allow gas exchange. Alternatively, N_2_ sparging was carried out under the same procedure, with sodium bicarbonate (at varying concentrations) added as the inorganic carbon source. Precultures were then inoculated into the medium at an initial OD_600_ of 0.1. Cultures were incubated at 37 °C with shaking at 200 rpm.

Rhamnose (16 mM) was supplied at the onset of cultivation, whereas arabinose (0.01%) and IPTG (20 μM) were added during the logarithmic growth phase, approximately 8 h postinoculation. The pH of the culture medium was adjusted to approximately 7 at 24, 36, 48, 60, 72, and 84 h by adding 2N NaOH.

### 2.3 High-Performance Liquid Chromatography (HPLC) Analysis

Glucose, rhamnose, formate, acetate, succinate, lactate, pyruvate, 1,2-propanediol, and ethanol were quantified by HPLC (Thermo Scientific Dionex Ultimate 3000 LC System) using Aminex HPX-87H column (300 × 7.8 mm, Bio-Rad) with 5 mM H_2_SO_4_ as the mobile phase at a flow rate of 0.6 mL/min and a column temperature of 45 °C. Detection of carbohydrate was performed with a refractive index detector (RID) and others were conducted with both a RID and a UV–vis detector. All samples were centrifuged for 5 min at 17,000*g* to remove the cells, and the supernatant was filtered using a 0.2 μm polyvinylidene difluoride (PVDF) filter. 10 μL of the sample was retrieved using an autosampler for the high-performance liquid chromatography (HPLC) analysis.^14^

### 2.4 LC-QTOF Analysis of Intracellular Metabolites and ^13^C-Tracing

Intracellular metabolites were extracted using a modified protocol.^15^ For bacterial culture, N_2_ sparging was carried out with ^13^C-labeled sodium bicarbonate at 144 mM added as the inorganic carbon source (as described in Section 2.2). A 2 mL cell suspension was filtered through a 3 mm filter (0.45 μm pore size, 13 mm diameter, PVDF), and the filter was immersed in 2.5 mL of extraction solvent (acetonitrile +0.1% formic acid/methanol/water, 40:40:20, v/v/v; precooled at −20 °C for 3 min), vortexed for 30 s, then transferred to another 50 mL tube containing 1.5 mL extraction solvent and vortexed again for 30 s. The two extracts were combined and divided into three 1.5 mL microtubes, centrifuged at 17,000*g* for 5 min at 4 °C, and the supernatants were collected into new 1.5 mL microtubes (three tubes). Samples were dried under vacuum at 25 °C, frozen at −80 °C overnight, and then lyophilized at −40 °C overnight. For extracellular metabolites, the culture broth was centrifuged at 17,000*g* for 10 min, and the supernatant was lyophilized.

For ^13^C-tracing, samples were analyzed using an Agilent 1290 UPLC coupled to a 6545B Q-TOF mass spectrometer. A BEH Amide column (2.1 × 100 mm, 1.7 μm) maintained at 35 °C was used. The mobile phase A consisted of 15 mM ammonium acetate and 0.3% ammonium hydroxide in water, and mobile phase B was 15 mM ammonium acetate and 0.3% ammonium hydroxide in 90% acetonitrile/5% water. The flow rate was 0.3 mL/min with an injection volume of 3 μL. The gradient started at 10% A and increased to 50% B by 8 min, maintained for 2 min and decreased to 10% in 1 min, maintained for 9 min.

Data were acquired in both positive and negative electrospray ionization modes (*m*/*z* 50–1200). The ion source was ESI with the following settings: sheath gas temperature 325 °C, sheath gas flow 10 L/min, nebulizer pressure 45 psi, drying gas temperature 280 °C, and drying gas flow 8 L/min. The capillary voltage was 3500 V, and the nozzle voltage was set at 500 V in positive mode and 1000 V in negative mode. The fragmentor voltage was 140 V. Data acquisition was performed in Auto MS/MS mode with a scan range of *m*/*z* 50–1200 and a scan speed of 2.5 spectra per second.

Metabolite signals and ^13^C-isotopologue distributions were processed using Agilent LC/MS Data Acquisition 11 and Agilent MassHunter Qualitative Analysis 10.0. Natural isotope abundance correction was applied using Agilent MassHunter Profinder 10, applying a 10 ppm mass tolerance and 0.6 min retention time window. Data were reported as isotopologue distributions or fractional enrichment.

### 2.5 Stoichiometry and Calculation

#### 2.5.1 Metabolism of *E. coli* BL21­(DE3) and Its Derivatives

1 $$glucose+2ADP+2{NAD}^{+}\to 2pyruvate+2ATP+2NADH$$

2 glucose+2ADP→2lactate+2ATP

3 $$L-rhamnose+ATP+NADH\to DHAP+1,2-propandiol+ADP+{NAD}^{+}$$

#### 2.5.2 Metabolism of Rubisco-Based Engineered *E. coli* ^8^

4 $$glucose+1.2{CO}_{2}\to 2.4pyruvate$$

5 $$glucose+2.4ADP+1.2{NAD}^{+}\to 2.4acetate+1.2formate+2.4ATP+1.2NADH$$

6 $$glucose+3.6NADH\to 2.4ethanol+1.2formate+3.6{NAD}^{+}$$

#### 2.5.3 Carbon Flux Analysis Using ^13^C Tracing

To determine the contribution of glycolysis versus the Rubisco-based engineered pathway, a carbon balance model was applied to eqs 1 and ([Disp-formula eq4])

eq 1 Represents conventional glycolysis, in which all carbon in pyruvate is derived from glucose. eq 4 represents the Rubisco-based engineered pathway, in which glucose and CO_2_ are coassimilated. Based on stoichiometry, 7.2 total carbon atoms are incorporated into 2.4 molecules of pyruvate, of which 1.2 carbon atoms (16.7%) originate from CO_2_. Thus, when ^13^C-labeled CO_2_ is supplied, the expected labeling in pyruvate from eq 4 is 16.7 atom %.

The fractional enrichment of pyruvate in ^13^C, denoted as y, was calculated from the isotopologue distribution according to

7 $$y=\frac{\sum_{i=0}^{n}m_{i}\cdot i}{\sum_{i=0}^{n}m_{i}\cdot n}$$

where *m*_*i*_ is the fractional abundance of the isotopologue with *i* labeled carbons and *n* = 3 for pyruvate. Thus, *y* represents the average fraction of carbons in the pyruvate pool that are ^13^C-labeled.

The observed isotopic enrichment of pyruvate was y atom % ^13^C. Let f_4_ denote the flux fraction through eq 4 and f_1_ = 1–f_4_ the flux through eq 1.

The labeling balance is expressed as

8 $$y=f_{4}\times 0.167$$

therefore, *f*_4_ will be

9 $$f_{4}=y/0.167$$

In addition to glucose metabolism, *l* -rhamnose was supplied as an inducer and was partially consumed as a carbon source. During rhamnose catabolism, *E. coli* generates DHAP, which re-enters central metabolism and can subsequently be converted to pyruvate (eq 3). Because rhamnose carbon is naturally unlabeled, the resulting pyruvate is also unlabeled and dilutes the ^13^C enrichment of the total pyruvate pool, leading to isotopic dilution bias in the measured labeling.

The contribution of rhamnose-derived pyruvate contribution (P_rha_, mM) can be estimated from rhamnose consumption using eq 3. One molecule of rhamnose (C6) produces one DHAP (C3), which can be converted to one pyruvate (C3), while the remaining C3 carbon converted into 1,2-propanediol. Accordingly, a DHAP-to-pyruvate conversion yield of 1:1 (mol/mol) was assumed for the fraction of rhamnose carbon entering central metabolism.

After accounting for unlabeled pyruvate originating from rhamnose, the expected ^13^C labeling in the mixed pyruvate pool becomes

$$y=f_{4}\times 0.167\times \frac{P_{glu}}{P_{glu}+P_{rha}}$$

10 $$=f_{4}\times 0.167\times \frac{{2n}_{glu}}{{2n}_{glu}+n_{rha}}$$

where *P*_glu_ (mM) is glucose-derived pyruvate, *n*_glu_ and *n*_rha_ denote the molar consumption of glucose and rhamnose, respectively.

Therefore

11 $$f_{4}=\frac{y}{0.167}\left(1+\frac{n_{rha}}{2n_{glu}}\right)$$

## 3 Results and Discussion

### 3.1 Engineering Glucose Metabolism Deficiency in *E. coli* through *zwf* Deletion and CRISPRi-Mediated *gapA* Repression

To achieve glucose metabolism deficiency in *E. coli*, *E. coli* MZ strain, a derivative of *E. coli* BL21­(DE3), in which the *zwf* gene was deleted to disrupt the oxidative pentose phosphate pathway (oPPP), was first employed. The *zwf* deletion not only block one of the glucose metabolism pathways, but also demonstrated that inactivation of the oPPP can effectively redirect carbon flux toward a Rubisco-based engineered pathway, enhancing its metabolic function.^7^

To suppress glycolysis to create a glucose metabolism deficiency, plasmid pSLiP-G2-RacE (abbreviated as pSLiP hereafter,^16^) carrying a CRISPR interference (CRISPRi) system targeting *gapA*,^17^ a key glycolytic gene, was introduced into *E. coli* MZ (Figure 1). As shown in Figure 2A, the control experiment of the MZ strain entered logarithmic growth at approximately 8 h and reached stationary phase after 36 h, with an OD_600_ of 0.68 ± 0.01. The strain MZ/pSLiP without dCas9 induction displayed a growth curve nearly identical to that of the parental MZ strain, suggesting negligible leaky expression of P_rha_ in the absence of l-rhamnose. Upon addition of l-rhamnose, MZ/pSLiP + rha exhibited significantly inhibited growth. At 36 h, the OD_600_ of MZ/pSLiP + rha was 0.30 ± 0.01, approximately 48% of that of the MZ/pSLiP strain. As shown in Figure 2B, both MZ and MZ/pSLiP strains consumed nearly all glucose (ca. 18 g/L) by 84 h, whereas the MZ/pSLiP + rha strain consumed only 8.3 ± 0.5 g/L. These findings confirm the functionality of pSLiP-G2-RacE, effectively conferring a glucose-metabolism-deficient phenotype.

**Figure 1:**
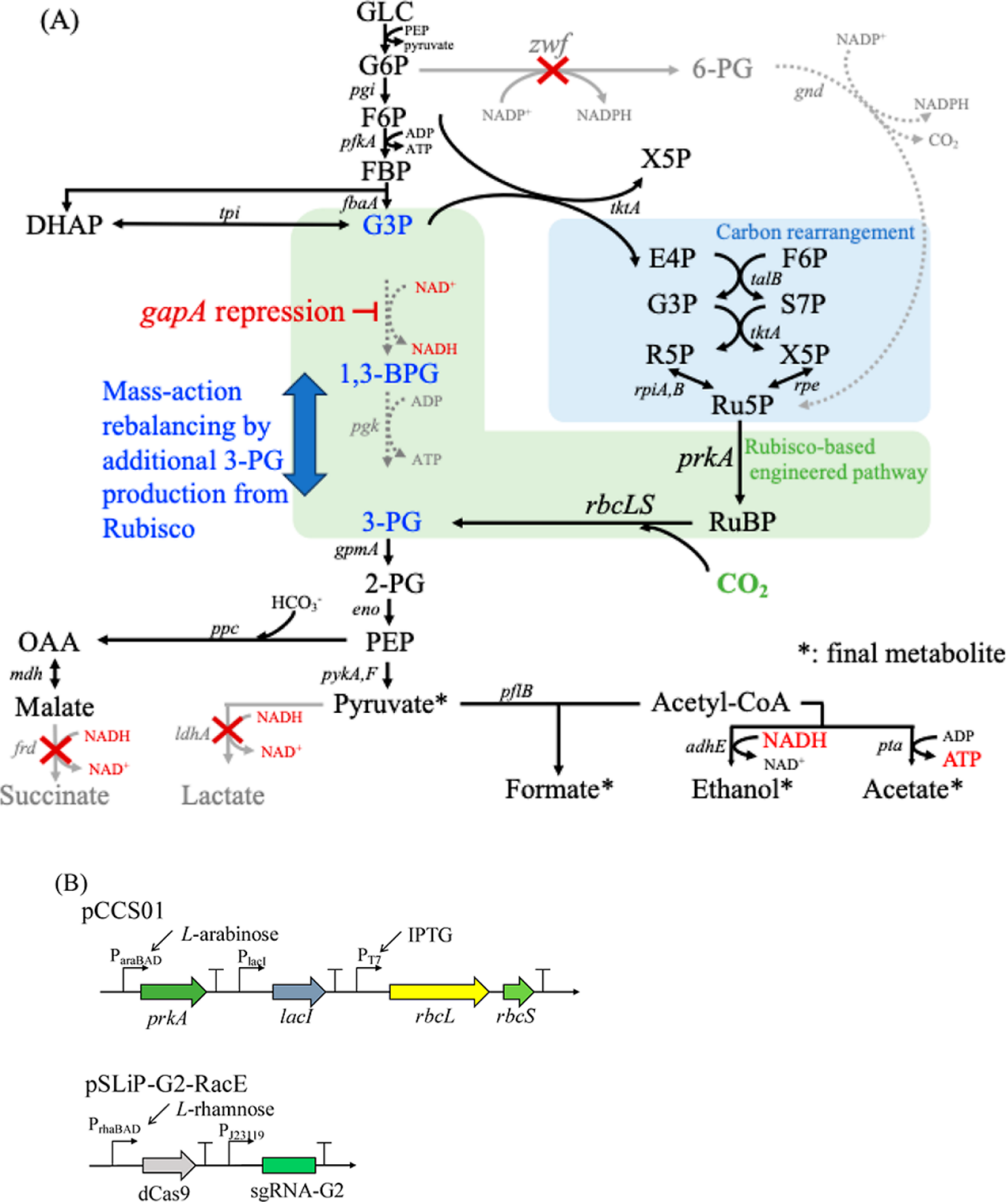
(A) Glucose metabolism can be restored in glucose metabolism deficiency *E. coli* by Rubisco-based engineered pathway, where the main metabolites are pyruvate, ethanol, acetate, and formate. Abbreviations: [GLC], glucose; [G6P], glucose 6-phosphate; [F6P], fructose 6-phosphate; [FBP], fructose 1,6-bisphosphate; [G3P], glyceraldehyde 3-phosphate; [DHAP], dihydroxyacetone phosphate; [1,3-BPG], 1,3-bisphosphoglycerate; [3 PG], 3-phosphoglycerate; [2 PG], 2-phosphoglycerate; [PEP], phosphoenolpyruvate; [OAA], oxaloacetate; [X5P], xylulose 5-phosphate; [E4P], erythrose 4-phosphate; [S7P], sedoheptulose 7-phosphate; [R5P], ribose 5-phosphate; [Ru5P], ribulose 5-phosphate; [RuBP], ribulose-1,5-bisphosphate. (B) Schematic diagram of the genetic circuits.

**Figure 2:**
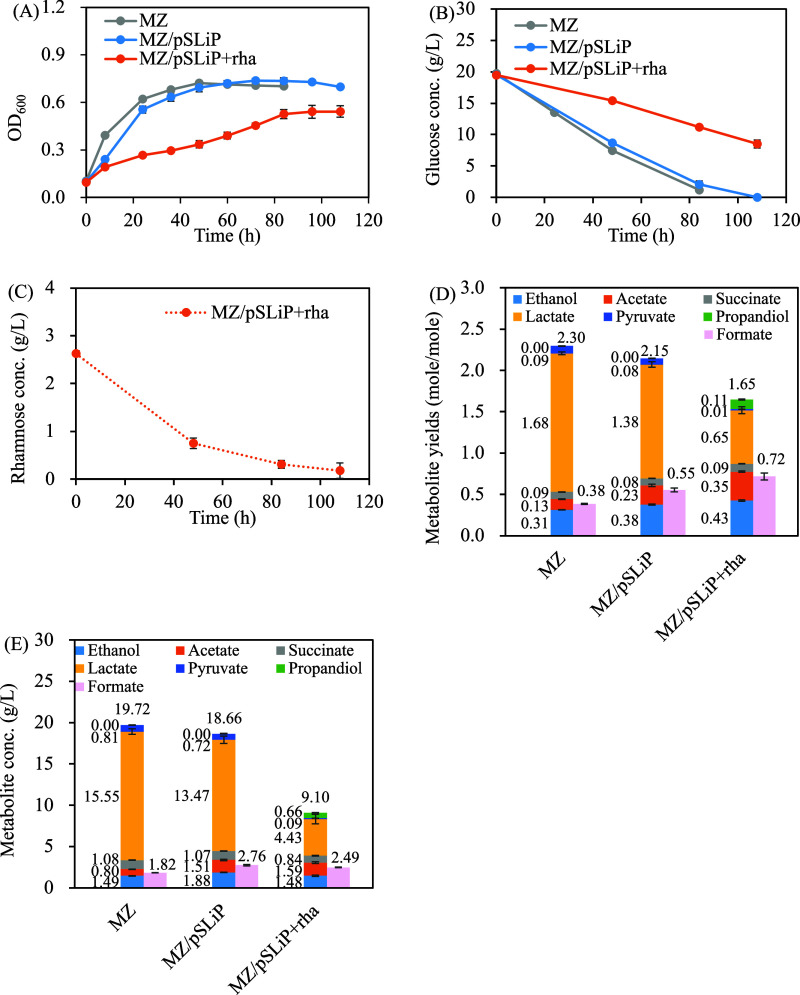
Effects of the CRISPRi-mediated *gapA* repression on *E. coli* strain MZ (*E. coli* BL21­(DE3) Δ*zwf*) under anaerobic conditions. (A) Growth curve, (B) glucose concentration profile, (C) rhamnose concentration profile, (D) metabolic yield, (E) metabolite concentration. The culture was cultivated under pure CO_2_ headspace. Data shown for 108 h. Data are expressed as mean ± standard deviation (*n* = 3, biological replicates).

Notably, a two-phase growth pattern was observed in the MZ/pSLiP + rha strain (Figure 2A). During the first phase (0–48 h), high rhamnose concentrations (Figure 2C) sustained strong CRISPRi activity, as evidenced by low glucose consumption (4.0 ± 0.2 g/L for MZ/pSLiP + rha vs 10.8 ± 0.4 g/L for MZ/pSLiP). To survive under restricted glucose metabolism, the strain utilized rhamnose as an alternative carbon source (Figure 2C). Once *l* -rhamnose was nearly exhausted, the second growth phase (48–108 h) commenced, characterized by weakened repression, resumed growth, and increased glucose consumption. This reversible repression effect was observed across strains tested (data not shown) and suggests that pSLiP-G2-RacE is a tunable and inducible tool for dynamic glycolytic control.

Previous study has shown the application of pSLiP-G2-RacE in *E. coli* strain WM335 under aerobic conditions in LB medium to repress glycolysis,^17^ It has reported nearly 100% transcriptional repression within the first 10 h of induction, with the inhibitory effect diminishing to ca. 30% by 20–24 h due to rhamnose depletion and metabolic adaptation.^17^ This study extends the application of pSLiP-G2-RacE to anaerobic conditions in a minimal defined medium. In contrast, under the more stringent anaerobic and nutrient-limited conditions used here, a significant level of residual glycolytic activity was observed in the MZ/pSLiP + rha strain. This residual activity may result from unknown metabolic adaptation in response to *gapA* repression. It is also important to note that the glucose-metabolism-deficient phenotype observed in MZ/pSLiP + rha is further attributed to the inherently weak activity of the Entner-Doudoroff (ED) pathway and the lack of the *gltP* gene, which encodes a proton symporter essential for gluconate uptake and ED pathway function.^13^

During rhamnose catabolism, *E. coli* generates dihydroxyacetone phosphate (DHAP), which reenters central metabolism, and extracellular 1,2-propanediol (eq 3,^18^). Figure 2D,E show a 1,2-propanediol production of 0.11 ± 0.01 mol/mol and 0.66 ± 0.01 g/L at 108 h, corresponding to a consumption of 2.5 ± 0.1 g/L rhamnose. This metabolic rerouting affects cellular redox balance and energy yields. Compared to glucose metabolism, rhamnose assimilation produced less NADH, reducing the demand for NADH-consuming pathways such as lactate and ethanol formation. Consequently, the lactate yield decreased whereas acetate production increased (Figure 2D,E) to maintain ATP supply. The elevated acetate suggests enhanced flux through the acetyl-CoA node, with ethanol acting as a redox-balancing metabolite. The overall decline in carbon recovery (Figure 2D) likely reflects suboptimal efficiency of the adapted glucose and small portion of *l* -rhamnose metabolism, where carbon may be lost to less efficient or undefined metabolic sinks. These findings reflect the evolutionary optimization of central carbon metabolism in *E. coli* for efficient carbon utilization.

### 3.2 Introduction of Rubisco-Based Engineered Pathway in Glucose Metabolism Deficiency *E. coli*

To restore glucose utilization in the glucose-metabolism-deficient strain MZ/pSLiP + rha strain, Rubisco-based engineered pathway (containing *prkA* and *rbsLS* genes in pCCS01) was introduced to test whether glucose can be reassimilated (Figure 1). Anaerobic cultivation was adopted to avoid the oxygenation that reduces overall carbon fixation efficiency. The anaerobic condition was achieved by flushing CO_2_ gas through the headspace of the serum bottle for 10 min. Besides, minimal M9 salt with 20 g/L glucose was used. The cultivation conditions are same as the ones used in Section 3.1.

As shown in Figure 3A, the strain MZ/pSLiP,pCCS01 exhibited improved growth and glucose consumption upon introduction of the Rubisco-based engineered pathway. The OD_600_ reached 0.85 ± 0.02 (Figure 3A), and glucose consumption reached 20.2 ± 0.1 g/L within 72 h (Figure 3B), indicating that the presence of pCCS01 enhanced both biomass accumulation and carbon utilization.

**Figure 3:**
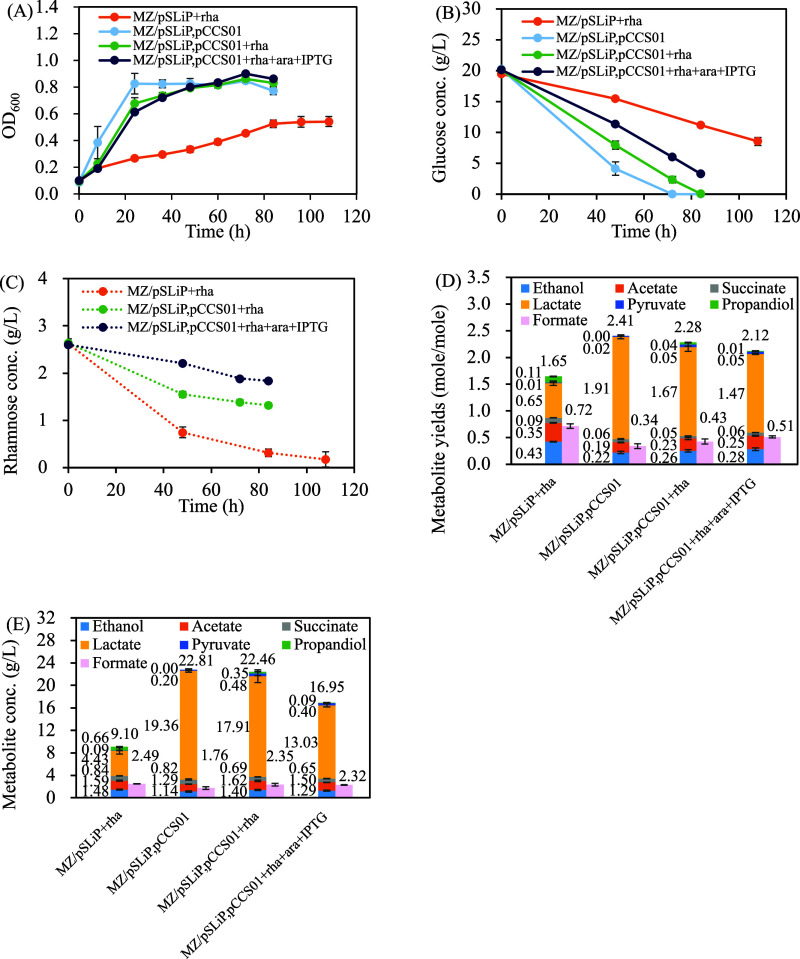
Introduction of the Rubisco-based engineered pathway (pCCS01) in glucose-metabolism-deficient *E. coli* MZ (*E. coli* BL21­(DE3) Δ*zwf*) under anaerobic conditions. (A) Growth curve, (B) glucose concentration profile, (C) rhamnose concentration profile, (D) metabolic yield, (E) metabolite concentration. The culture was cultivated under pure CO_2_ headspace. Data shown for 84 h. Data are expressed as mean ± standard deviation (*n* = 3, biological replicates).

Upon addition of rhamnose to induce CRISPRi-mediated *gapA* repression (MZ/pSLiP,pCCS01+rha), both growth and glucose consumption declined moderately, confirming that glycolysis was partially repressed. Nevertheless, glucose consumption remained high, with approximately 20 g/L utilized by 84 h. When *l* -arabinose and IPTG were added to actively induce the Rubisco-based engineered pathway in MZ/pSLiP,pCCS01+rha + ara + IPTG, the trend toward low glucose consumption was observed, yet 16.8 ± 0.2 g/L can still be consumed by 84 h, in contrast to only 8.3 ± 0.5 g/L consumed by MZ/pSLiP + rha.

Figure 3C demonstrates that in MZ harboring pSLiP, a pronounced deficiency in glucose metabolism in the strain MZ/pSLiP + rha was accompanied with the consumption *l* -rhamnose. In contrast, the introduction of the plasmid pCCS01 into MZ/pSLiP,pCCS01+rha resulted in vigorous glucose consumption, which results in mild consumption of *l* -rhamnose. This shift suggests that the Rubisco-based engineered pathway encoded by pCCS01 is functionally active and capable of restoring glucose metabolism.

Consistent with the enhanced glucose uptake of MZ/pSLiP,pCCS01, total metabolite yields increased and reached 2.4 ± 0.1 mol/mol with a high lactate production of 1.91 ± 0.03 mol/mol or 19.4 ± 0.3 g/L (Figure 3D,E). Upon glycolysis repression by rhamnose induction in MZ/pSLiP,pCCS01+rha, the lactate yield decreased to 1.67 ± 0.08 mol/mol glucose, while pyruvate appeared and accumulated to 0.05 ± 0.03 mol/mol. Notably, the levels of acetate and ethanol (C-2 products) also increased in MZ/pSLiP,pCCS01+rha. Further induction of the Rubisco-based pathway via arabinose and IPTG (MZ/pSLiP,pCCS01+rha + ara + IPTG) preserved the altered metabolite profile, including increased C-2 compound production and pyruvate accumulation, along with even lower lactate yield of 1.47 ± 0.01 mol/mol (Figure 3D) and 13.0 ± 0.3 g/L (Figure 3E). The change toward high pyruvate and C-2 compounds upon the introduction of Rubisco-based engineered pathway suggests a partial diversion of carbon toward the engineered pathway, yet glycolysis is suggested to be the primary route for glucose catabolism.^19^

Introducing the Rubisco-based pathway appears to synergistically enhance glucose uptake, plausibly by increasing the cellular ATP demand: carbon is diverted into a zero-ATP-yield Rubisco-based engineered pathway, which, in turn, stimulates glycolytic flux.^20^ The enhanced glucose consumption also suggests that native glycolytic control was effectively bypassed; accordingly, MZ/pSLiP,pCCS01 exhibited a high specific glucose consumption rate and functioned as a high-efficiency whole-cell catalyst. This behavior illustrates a cooperative coupling between glycolysis and the engineered carbon fixation pathway in driving robust growth and carbon utilization. Nevertheless, the increased glucose consumption and total metabolite output of MZ/pSLiP,pCCS01 are attributed largely to glycolysis. As shown previously,^19^ metabolic engineering leads to a shift toward pyruvate accumulation, and isotopic labeling in this study further supports this interpretation (see below). Lactate remained the predominant end product, indicating that glycolysis still dominates overall carbon partitioning under these conditions, as lactate formation serves to balance the reducing equivalents generated during glycolysis.

Notably, the apparent coordination between glycolysis and the Calvin cycle may depend on the basal expression level of pCCS01. A low (leaky) level of Prk/Rubisco activity could be sufficient to support partial Calvin cycle flux while maintaining robust glycolytic throughput. In this regime, Rubisco generates 3 PG, which enters central metabolism downstream of the *gapA*-controlled step, thereby partially restoring carbon continuity around the G3*P*/1,3-BPG bottleneck and allowing glycolysis-derived energy metabolism to proceed. In contrast, strong induction of the CBB module appears to reduce glucose consumption (Figure 3B), suggesting that excessive pathway expression may impose additional energetic and regulatory burdens. For example, increased ATP demand from PrkA, altered metabolite pools, or imbalance arising from RuBP accumulation and redox stress could decrease overall glucose uptake and weaken the coordinated state.

### 3.3 Blocking NADH-Reoxidation Sinks Effectively Increases the Glycolytic Activity and Provides an Unique Metabolite Profile

In MZ/pSLiP,pCCS01 + rha + ara + IPTG, the Rubisco-based engineered pathway is arguably active but conventional glycolysis remains dominant. We hypothesized that low CO_2_ fixation results from the highly compatibility of glycolysis with endogenous NAD^+^/NADH-balancing routes (*ldhA* and *frd*), allowing glycolysis to outcompete the engineered branch. Consistent with the theoretical stoichiometries,^8^ introducing the Rubisco-based engineered pathway produced a metabolite shift toward high pyruvate and C-2 products. In eq 4, pyruvate is produced by combining 1 mol glucose and 1.2 mol of CO_2_ with no net bioenergetic concerns. ATP for growth is then supplied by substrate-level phosphorylation via the Pta-AckA acetate pathway (eq 5). Under *ldhA* ^+^ conditions, lactate formation competes with acetate for NADH reoxidation. Since glycolysis and *ldhA* is highly compatible, the minimal acetate production weakens the competitiveness of the Rubisco-based engineered pathway. We therefore removed the principal NADH sinks to favor acetate-coupled ATP generation: Δ*ldhA* (strain MZL) and Δ*ldhA* Δ*frd* (strain MZLF), in combination with CRISPRi-mediated *gapA* repression to further limit glycolysis.

Figure 4 shows that MZL had a retarded growth and reached OD_600_ of 0.56 ± 0.01 at and beyond 48 h. The total glucose consumption of MZL was 9.0 ± 0.1 g/L in 84 h, ca. 8 g/L less than the parental strain MZ. When *gapA* was repressed, the growth barely reached 0.33 ± 0.01 at 48 h (Figure 4) with a lower glucose consumption of 7.3 ± 0.1 g/L at 48 h (Figure S1A). Activating the Rubisco-based engineered pathway­(MZL/pSLiP,pCCS01 + rha + ara + IPTG) improved growth and redirected products toward pyruvate and acetate (Figure S1B,C). However, MZL grew better than MZL/pSLiP,pCCS01 + rha + ara + IPTG.

**Figure 4:**
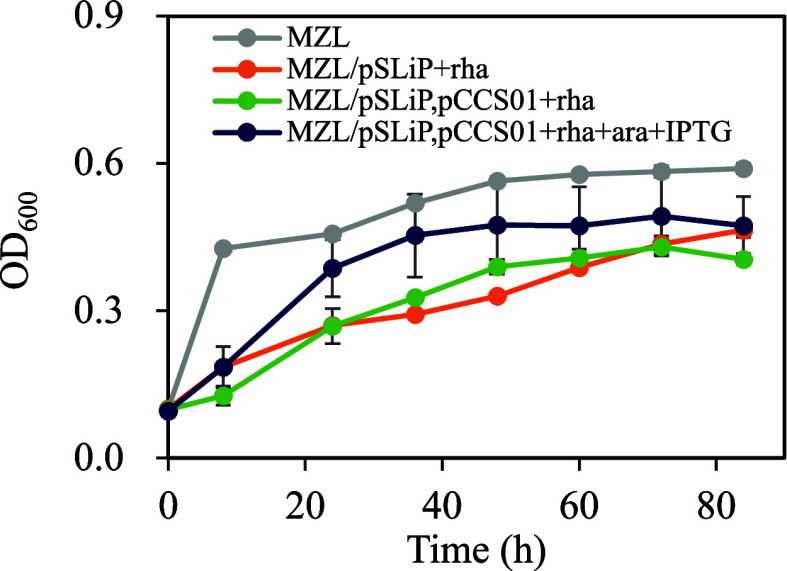
Growth curves of glucose-metabolism-deficient *E. coli* MZL (BL21­(DE3) Δ*zwf* Δ*ldhA*) carrying the Rubisco-based engineered pathway (pCCS01) under anaerobic conditions. The culture was cultivated under pure CO_2_ headspace. Data shown for 84 h. Data are expressed as mean ± standard deviation (*n* = 3, biological replicates).

Additional deletion of *frd* in MZL yielded MZLF, which exhibited low growth (OD_600_ = 0.42 ± 0.01, Figure 5A) and the lowest glucose consumption among all strains (Figure 5B), demonstrating that removing both NADH sinks severely truncates glycolysis. Upon introducing the Rubisco-based engineered pathway restored glucose utilization. MZLF/pSLiP,pCCS01 + rha grew 30–70% higher OD_600_ than MZLF/pSLiP + rha; with active induction of PrkA and Rubisco (MZLF/pSLiP,pCCS01 + rha + ara + IPTG), OD_600_ improved by 50–90%. Glucose consumption was also markedly improved. While MZLF/pSLiP + rha consumed only 7 g/L glucose by 84 h, the strain harboring the Rubisco-based engineered pathway (MZLF/pSLiP,pCCS01 + rha + ara + IPTG) exhausted the same amount within just 48 h, and reached a total consumption of 14 g/L by 84 h, demonstrating a significant recovery in glucose metabolic capacity.

**Figure 5:**
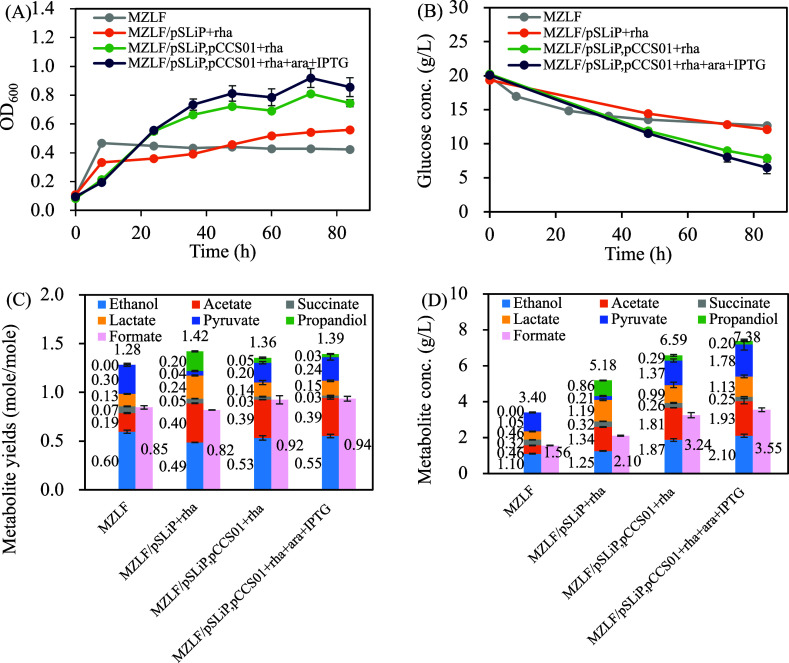
Introduction of the Rubisco-based engineered pathway (pCCS01) in glucose-metabolism-deficient *E. coli* MZLF (*E. coli* BL21­(DE3) Δ*zwf* Δ*ldhA*Δ*frd*) under anaerobic conditions. (A) Growth curve, (B) glucose concentration profile, (C) metabolic yield, (D) metabolite concentration. The culture was cultivated under pure CO_2_ headspace. Data shown for 84 h. Data are expressed as mean ± standard deviation (*n* = 3, biological replicates).

Figure 5C,D further illustrate that both MZLF/pSLiP,pCCS01+rha and MZLF/pSLiP,pCCS01 + rha + ara + IPTG exhibited high pyruvate and C-2 products, indicating a distinct and consistent shift in fermentation product distribution. The total metabolite concentration increased from 5.18 g/L to 7.21 g/L (Figure 5D). These data suggest that the *E. coli* strain harboring Rubisco-based engineered pathway as a central metabolism is characterized by high pyruvate and C-2 products compared to nonengineered controls. Notably, robust induction of PrkA/Rubisco boosted performance in the MZLF background but not in MZ (compare Figure 5 vs Figure 3), indicating that higher-level regulation can mask pathway gains unless competing redox circuits are removed.

Collectively, these results show that blocking NADH-balancing routes (*ldhA*, *frd*) and constraining glycolysis create a dependency that the Rubisco-based engineered pathway can exploit, thereby restoring glucose uptake, shifting redox allocation toward acetate-linked ATP production, and establishing a distinctive, pyruvate/C2-biased fermentation phenotype.

### 3.4 CO_2_-Dependnet Pyruvate Production in *E. coli* MZLF/pSLiP,pCCS01 + rha + ara + IPTG

A bicarbonate dose-dependent experiment was conducted using the Rubisco-based engineered *E. coli* MZLF/pSLiP,pCCS01 + rha + ara + IPTG. Cultures were grown in sealed serum bottles filled with an N_2_ headspace (replacing CO_2_ used in prior sections) and supplemented with sodium bicarbonate at 12, 48, 96, and 144 mM.

Both metabolite yield (Figure 6A) and total metabolite concentration (Figure 6B) increased with bicarbonate concentration. The product distribution matched CO_2_-headspace experiments, featuring high pyruvate and C-2 products. Notably, pyruvate production positively correlated with bicarbonate in strains MZLF/pSLiP,pCCS01 + rha + ara + IPTG and MZ/pSLiP,pCCS01 + rha + ara + IPTG whereas wild-type *E. coli* BL21­(DE3) had no pyruvate production under the same bicarbonate concentrations, indicating that bicarbonate specifically supports activity of the Rubisco-based engineered pathway.

**Figure 6:**
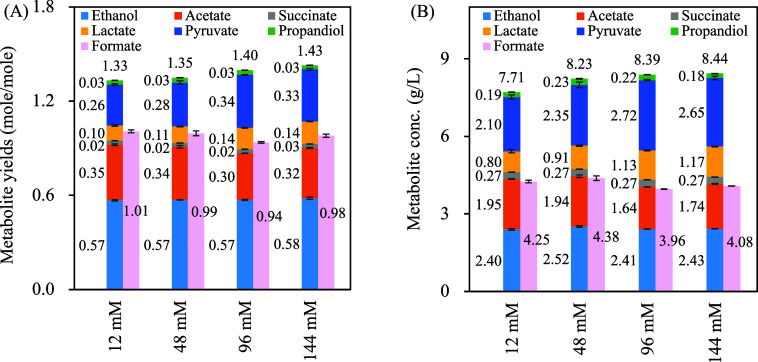
(A) Metabolite yields and (B) total metabolite concentrations in bicarbonate-dependent fermentation of *E. coli* MZLF/pSLiP,pCCS01 + rha + ara + IPTG. Data are expressed as mean ± standard deviation (*n* = 3, biological replicates).

Bicarbonate supplementation modestly promoted growth, (achieving OD_600_ above 2, Figure S2D), whereas cultivation under a pure CO_2_ headspace slightly inhibited growth (OD_600_ below 1, Figure 5A), suggesting that elevated pCO_2_ imposes physiological stress.

### 3.5 Isotope Profile of the Central Metabolic Pathway in *E. coli* MZLF/pSLiP,pCCS01 + rha + ara + IPTG

The isotopic fractional enrichment profile shows that the central metabolism could be divided into two regimes, with pyruvate as a soft boundary: i.e., glucose metabolism upstream and the tricarboxylic acid (TCA) cycle downstream. Upstream of pyruvate, all detected sugar phosphates have relatively low fractional enrichment of 0.6–1.4%. On the other hand, the TCA cycle has extensive fractional enrichment, approaching 30% (Figure 7). This dichotomy was further supported by the labeling patterns of amino acids. Amino acids derived from glycolytic intermediates such as 3 PG, PEP, and pyruvate displayed fractional enrichment of 1–3%, consistent with sugar phosphates, whereas, amino acids derived from the TCA cycle exhibited high fractional enrichment.

**Figure 7:**
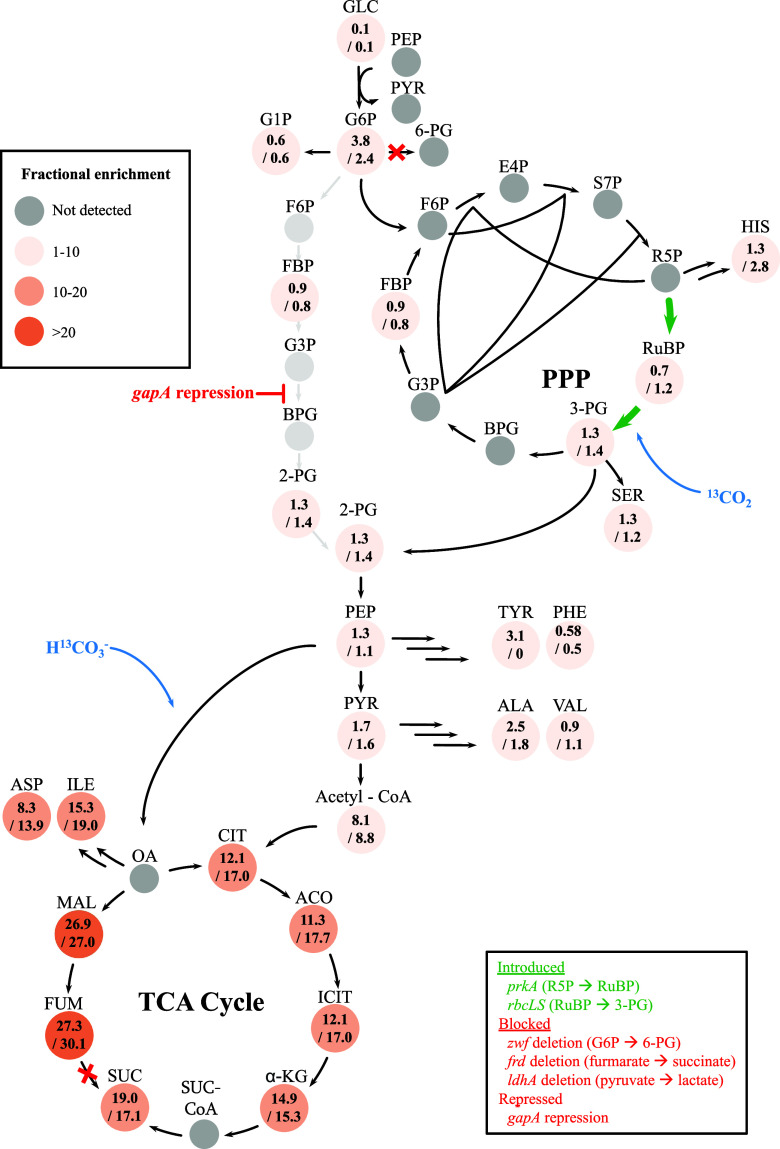
Isotopic labeling analysis showing two entry points of CO_2_ in *E. coli* MZLF/pSLiP,pCCS01 + rha + ara + IPTG. Fractional enrichment of metabolites at 12 and 24 h is shown. Values represent the mean of three biological replicates.

These distinct labeling patterns indicate that ^13^C labeled CO_2_/HCO_3_^–^ enters central metabolism through two main routes: Rubisco and PEP carboxylase. Two lines of evidence support Rubisco as a major entry point. First, both the substrate (RuBP) and product (3 PG) of Rubisco were detected by LC-QTOF and 3 PG was found to be labeled at both 12 and 24 h (Figure 7). Second, pyruvate, the net product of the Rubisco-based engineered pathway, was labeled with fractional enrichment of 1.7 ± 0.3 and 1.6 ± 0.0 at 12 and 24 h, respectively, exclusively in the *m* + 1 isotopologue. The quantitative labeling gap between sugar phosphate and TCA cycle suggests that ^13^C label in pyruvate was not derived from anaplerosis, further supporting CO_2_ fixation through the Rubisco-based engineered pathway.

One unexpected result is that the Calvin cycle is reconstituted in *E. coli* and coexisted with glycolysis. The reconstitution of the Calvin cycle can be evidenced by the labeling of the intermediates of FBP, whereas the non-Calvin cycle intermediates, i.e., G6P and G1P had relatively lower fractional enrichment of less than 0.6%. While the reconstitution of the Calvin cycle is consistent with previous study by expressing PrkA and Rubisco,^4^ this study showed that the Calvin cycle can coexist with glycolysis, more specifically, the Calvin cycle can enhance the glycolytic activity that has never reported before (Figures 3B and 5B). This enhancement could arise from mass-action rebalancing at the shared 3PG node. More specifically, Rubisco directly generates 3PG downstream of the GAPDH step, restoring the G3*P*/3PG balance and allowing flux to pass through this near-equilibrium reaction even at reduced GapA levels.^21^ Consequently, carbon enters lower glycolysis from both glucose catabolism and CO_2_ fixation. Rather than bypassing glycolysis, the Calvin cycle provides an additional entry point at 3PG that relieves the limitation in the GAPDH-repressed G3*P*/3PG conversion and increases overall glycolytic throughput. Notably, only a modest Rubisco-derived 3PG flux (10%) is required to restore transmission across this near-equilibrium step. At higher activities, however, excess 3PG production would be expected to promote reverse flux through the reversible GAPDH reaction, leading to accumulation of upper-glycolytic intermediates and reduced glycolysis. Thus, the beneficial effect arises from partial compensation of the GAPDH limitation rather than maximal carbon fixation flux. Together, these results indicate that the engineered Calvin cycle functions as a metabolic buffering module at the 3PG node, reinforcing glycolytic throughput by supplying downstream intermediates within an optimal flux range. The observed phenomenon arises from an inhibition experiment rather than a gene knockout.

The glycolytic flux through the Rubisco-based engineered pathway has previously been suggested to be estimated by the fractional enrichment of 3 PG.^22^ In our study, however, both the substrate (RuBP) and the product (3 PG) of Rubisco were labeled to a similar extent, making the 3 PG-based approach unsuitable. Instead, pyruvate labeling was used as the readout, since pyruvate is the net product of the Rubisco-based engineered pathway and its theoretical maximum enrichment is 16.7 atom %. According to eq 11, the flux fraction through the Rubisco-based engineered pathway depends on both the measured pyruvate enrichment and the ratio of rhamnose to glucose consumption. Using the measured pyruvate enrichment of 1.6–1.7%, together with the glucose consumption of 14.3 ± 0.1 g L^–1^ h^–1^ and rhamnose consumption of 1.1 ± 0.1 g L^–1^ h^–1^ (data not shown), the calculated flux fraction f_4_ is approximately 10%, indicating that about 10% of glucose was metabolized through the Rubisco-based engineered pathway. Note that the dilution bias caused by rhamnose-derived carbon is small and does not significantly affect the interpretation of the metabolic flux distribution. The average glucose consumption rate of *E. coli* MZLF/pSLiP,pCCS01 + rha + ara + IPTG over 84 h was 170 ± 1 mg L^–1^ h^–1^, of which 17.0 ± 0.1 mg L^–1^ h^–1^, 10% of total glucose uptake, was directed through the Rubisco pathway. Using eq 4, the corresponding CO_2_ fixation rate was estimated at 5.0 ± 0.1 mg L^–1^ h^–1^.

Finally, the labeling profiles of amino acids derived from 3 PG, PEP, and pyruvate provide direct evidence of mixotrophic growth, integrating both glucose metabolism and CO_2_ fixation. The final products are pyruvate, acetate, ethanol, and formate. Note that acetate has been recently proposed as a promising feedstock for biobased chemical production.^23–25^

## 4 Discussion

In nature, metabolism can be broadly classified as autotrophic or heterotrophic, each conferring distinct evolutionary advantages. Autotrophs fix inorganic carbon, while heterotrophs consume organic carbon; together, they often form mutualistic associations that enhance ecological resilience. Mixotrophs combine these two strategies, functioning both as primary producers through photosynthesis and as consumers through prey ingestion. This dual role stabilizes nutrient ratios (C:N:P) and increases food-web flexibility.^26^ From an engineering perspective, mixotrophs offer similar versatility: their metabolic modes can be switched on or off to balance growth and product synthesis. For instance, in *Cupriavidus necator* growing on palm oil, activation of the CBB cycle recycles surplus reducing power, restores redox balance, improves metabolic efficiency, and enhances polyhydroxyalkanoate (PHA) production.^27^ Because the network converges at the pyruvate and acetyl-CoA nodes, this configuration is particularly suitable for the production of pyruvate-derived and acetyl-CoA-derived compounds, such as organic acids, alcohols,^12,28,29^ and polyhydroxyalkanoates.^30^ In such pathways, carbon loss through decarboxylation is often unavoidable; incorporation of a functional Calvin cycle provides a route to reassimilate released CO_2_ and potentially improve product yield. Moreover, this coordination strategy could be integrated with the serine shunt,^31^ engineered phosphoketolase pathways.^32–34^

This study demonstrates that synthetic mixotrophy can be established in *E. coli* by deliberately overcoming the evolutionary dominance of glycolysis. Robust central metabolism requires alignment with both energy and redox balance; thus, engineering strategies must consider not only gene additions or deletions but also auxiliary pathways. In metabolic engineering terms, central metabolism can be rebuilt by introducing optimal balancing modules, such as ATP homeostasis^35^ or NAD^+^/NADH regulation. Here, stoichiometric analysis revealed that disruption of NAD^+^/NADH balancing pathways attenuates and even deactivates glycolysis in MZLF under anaerobic conditions with glucose as the sole carbon source. Against this metabolic background, it became feasible to introduce an evolutionarily less competitive but engineering-oriented pathway: the Rubisco module. This shift enabled *E. coli* MZLF/pSLiP,pCCS01 + rha to sustain growth and glucose consumption via glycolysis/Calvin_cycle. Notably, the ED pathway cannot compensate for *gapA* deletion because it generates G3P, which still requires GapA for further metabolism; thus, repression of *gapA* blocks both EMP flux and any potential bypass via ED. In addition, the ED pathway provides insufficient ATP to support the ATP-consuming Calvin cycle reactions and cannot effectively sustain Ru5P/RuBP regeneration; notably, glucose consumption was even increased (Figure 3B). Finally, the ED pathway has been reported to exhibit only weak activity in *E. coli*.^13^ Therefore, a Calvin cycle-ED configuration is unlikely to become dominant.

Energy balance plays a critical role in maintaining this rewired metabolism. When pyruvate is produced through the Rubisco-based engineered pathway, no ATP is generated, creating a demand for ATP synthesis through the acetyl-CoA node leading to acetate formation. In parallel, the acetyl-CoA node leading to ethanol acts as a buffering mechanism to maintain redox balance. According to the stoichiometries (eqs 1–3), Rubisco-based *E. coli* assimilates glucose and CO_2_ into pyruvate, which is further directed into acetate, ethanol, and formate without carbon loss. As a result, this strain achieves net positive CO_2_ assimilation and accumulates pyruvate, acetate, ethanol, and formate as the major end products. Notably, mixotrophic growth conferred faster growth rates than strict autotrophy, underscoring the advantage of this engineering design.

The coexistence of glycolysis and the Calvin cycle provides the advantage of high glycolytic flux, yet this evolutionary benefit depends on downstream redox-balancing pathways. While glycolysis alone ensures substrate-level phosphorylation and redox balancing by lactate production, coupling it with the Calvin cycle imposes an additional requirement for C2-derived metabolites to fulfill these roles. This engineered coexistence of glycolysis and the Calvin cycle mirrors lithotrophic lifestyles such as that of *C. necator*, where multiple pathways jointly secure both metabolic robustness and evolutionary adaptability.^30^

This study demonstrates that reconstruction of central metabolism does not necessarily compromise cellular fitness. When grown under identical conditions, MZLF/pSLiP,pCCS01 + rha + ara + IPTG exhibited slower growth than BL21­(DE3) but ultimately reached a higher final OD_600_ (Figure S2). Further optimization could be done by cell-free systems that have been demonstrated as a means to accelerate pathway prototyping.^36^ Finally, multiplex experimentation integrating genotypic variation with metabolic profiling can elucidate key features of metabolic networks, thereby constituting a structured resource for artificial intelligence-driven analysis and modeling.^37^

Previous studies have reported that glycolate/glyoxylate can arise from Rubisco-mediated oxygenation and may serve as useful chemical precursors.^38,39^ Therefore, the coexistence of glycolysis and the Rubisco-based engineered pathway under aerobic conditions may offer a potential route for glycolate/glyoxylate production, with ATP supplied by the electron transport chain. More detailed investigation of NADH oxidation, ATP generation, and product formation under oxygenated conditions will be pursued in future work.

Although the Rubisco-based engineered pathway was functional under the conditions tested, its performance is likely constrained by the low catalytic turnover rate of Rubisco and the ATP demand associated with PrkA activity. Furthermore, efficient operation of the pathway depends on appropriate expression, folding, and stoichiometric balance of the heterologous enzymes; suboptimal expression levels or protein burden may limit effective flux through the pathway. The absence of dedicated carbon-concentrating mechanisms in *E. coli* may further restrict carboxylation efficiency. Future work could therefore focus on optimizing enzyme expression and folding, improving RuBP regeneration, and enhancing intracellular carbon availability^40^ to improve pathway performance.

## Supplementary Material



## Notes

Metabolism of glucose-metabolism-deficient *E. coli* MZL (*E. coli* BL21­(DE3) Δ*zwf* Δ*ldhA*) harboring Rubisco-based engineered pathway (pCCS01) under anaerobic conditions (Figure S1); Growth profile of *E. coli* BL21­(DE3) and MZLF/pSLiC,pCCS01+rha + ara + IPTG in anaerobic minimal medium supplemented with NaHCO_3_ (Figure. S2) (PDF)
