# Supplementary material for: Coordinating Calvin Cycle and Glycolysis in Escherichia coli
Source: ACS Synth Biol. 2026 Mar 26;15(4):1467–79. doi: 10.1021/acssynbio.5c00854 (PMC13097247; doi:10.1021/acssynbio.5c00854)
Supplement: Supplementary file 1 [file sb5c00854_si_001.pdf]

Supporting Information for Publication

Coordinating Calvin cycle and Glycolysis in *Escherichia coli*

Yu-Jen Lin, Hsien-Tse Chen, Pei-Yi Lin, Yi-Jyun Lai, and Si-Yu Li*

Department of Chemical Engineering, National Chung Hsing University, Taichung 402, Taiwan

* Email: syli@dragon.nchu.edu.tw

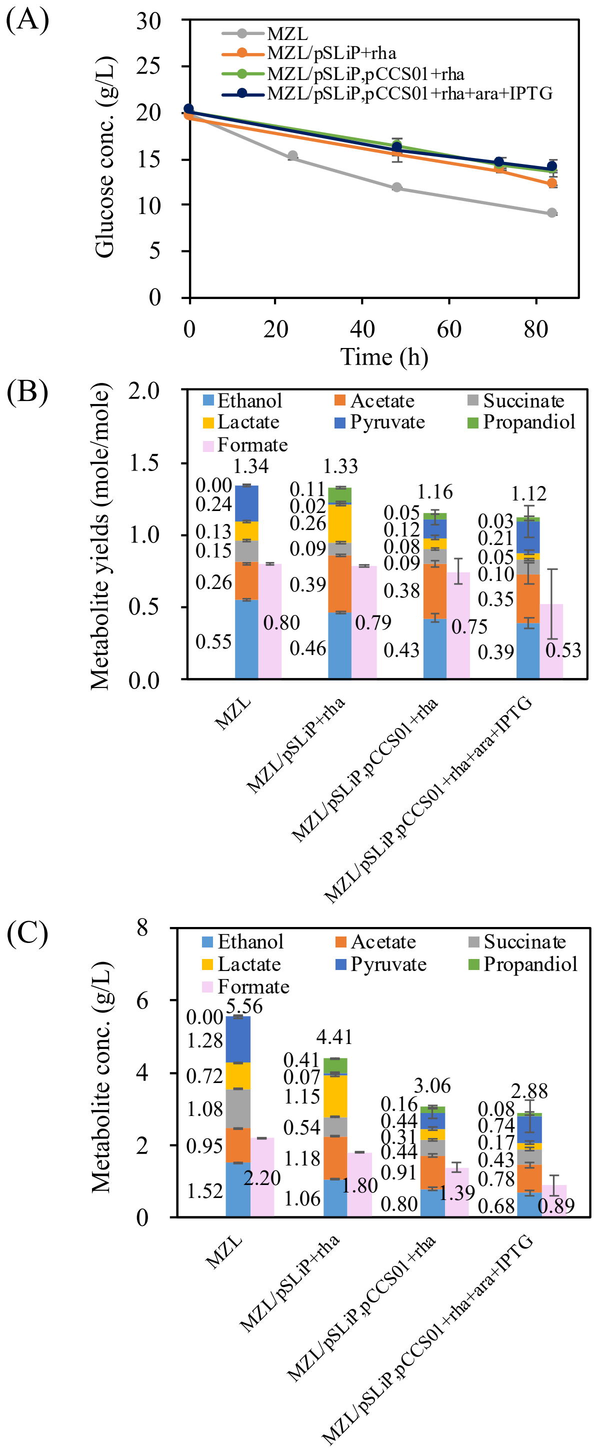

Figure S1. Introduction of a Rubisco-based engineered pathway (pCCS01) in glucose-metabolism-deficient *E. coli* MZL (*E. coli* BL21(DE3) Δ*zwf* Δ*ldhA*) under anaerobic conditions. (A) Glucose concentration profile, (B) metabolic yield, (C) metabolite concentration. Data shown for 84 h. Data are expressed as mean ± standard deviation (n=3, biological replicates).

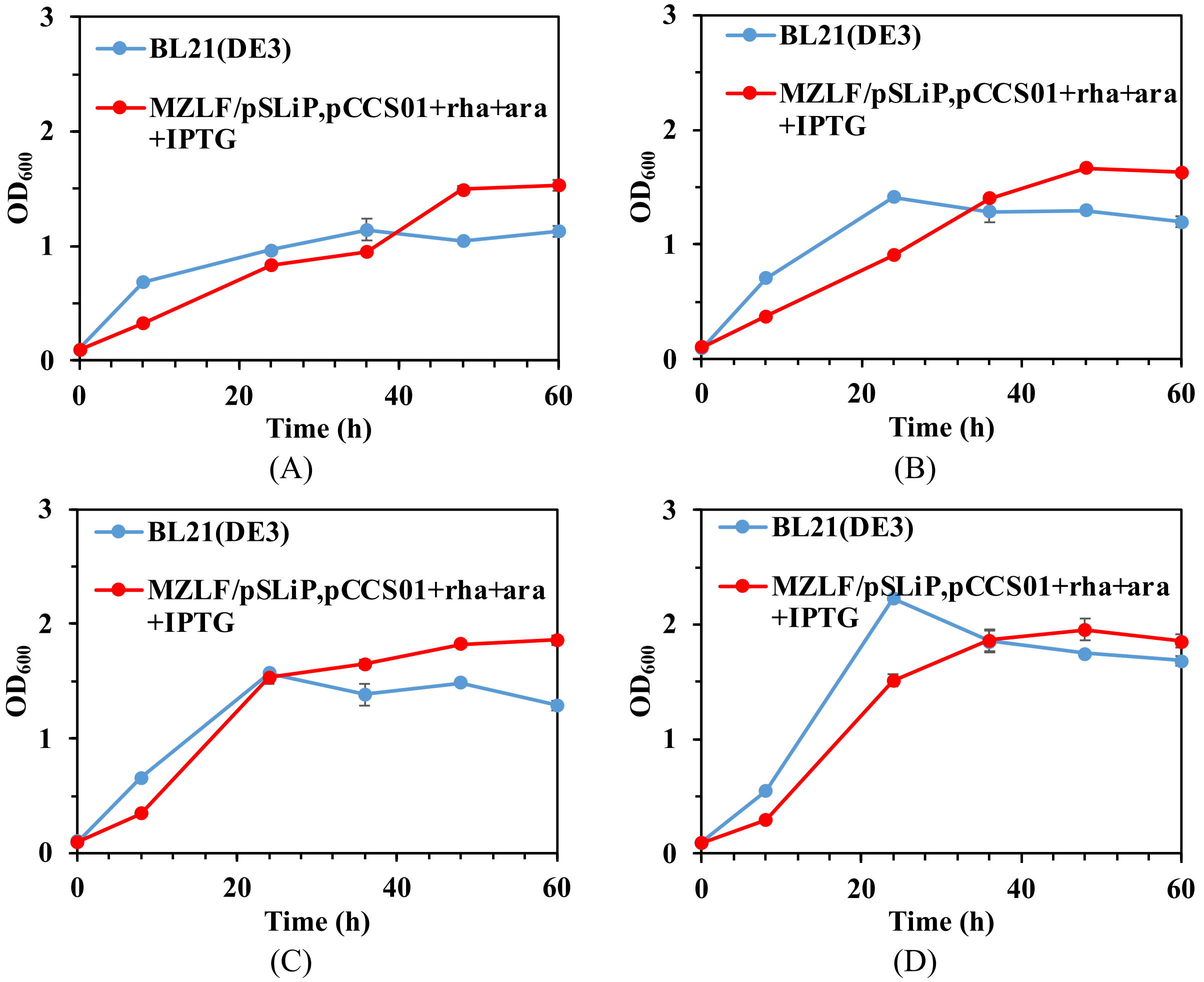

Figure S2. Growth profile of *E. coli* BL21(DE3) and MZLF/pSLiC,pCCS01+rha+ara+IPTG in anaerobic minimal medium supplemented with $NaHCO_3$ at (A) 12, (B) 48, (C) 96, or (D) 144 mM. Data are expressed as mean ± standard deviation (n=3, biological replicates).
